# IgG subclass responses to excreted-secreted antigens of *Plasmodium falciparum* in a low-transmission malaria area of the Peruvian Amazon

**DOI:** 10.1186/s12936-018-2471-6

**Published:** 2018-09-11

**Authors:** Rafael Saavedra-Langer, Jorge Marapara, Andree Valle-Campos, Salomón Durand, Maria E. Vásquez-Chasnamote, Hermann Silva, Viviana Pinedo-Cancino

**Affiliations:** 1 Fundación para el Desarrollo Sostenible de la Amazonía Baja del Perú, Universidad Nacional de la Amazonía Peruana, Iquitos, Perú; 2 Laboratorio de Investigación de Productos Naturales Antiparasitarios de la Amazonía Peruana, Centro de Investigación de Recursos Naturales de la Amazonía, Universidad Nacional de la Amazonía Peruana, Iquitos, Perú; 3 Centro de Investigación en Enfermedades Tropicales “Maxime Kuczynski”, Instituto Nacional de Salud, Lima, Perú; 4 Facultad de Medicina Humana, Universidad Nacional de la Amazonía Peruana, Iquitos, Perú

**Keywords:** ELISA, Exoantigens, Antibodies, Asymptomatic, Zungarococha

## Abstract

**Background:**

Malaria in Peru is concentrated in the Amazon region, especially in Loreto, and transmission is focused in rural and peri-urban communities. The government has approved a malaria elimination plan with a community approach and seeks to reduce the risk of transmission through preventive interventions, but asymptomatic and low-parasite-density infections are challenges for disease control and elimination. IgG antibodies play a critical role in combating infection through their ability to reduce parasitaemia and clinical symptoms. In particular, IgG subclasses have important roles in controlling malaria disease and may provide new insight into the development of malaria control strategies and understanding of malaria transmission. Through the use of excreted-secreted antigens from *Plasmodium falciparum*, were evaluated the responses of the four IgG subclasses in symptomatic and asymptomatic malarial infections.

**Results:**

Higher levels of whole IgG were observed in asymptomatic carriers (P < 0.05). IgG3 and IgG1 were the most prevalent subclasses and did not show differences in their antibody levels in either type of carrier. All symptomatic carriers were positive for IgG4, and the presence of IgG3 and IgG2 were correlated with protection against parasitaemia. IgG2 showed lower prevalence and antibody titers in comparison to other subclasses.

**Conclusions:**

This is the first study that characterizes the IgG subclass response in the Peruvian Amazon, and these results show that even in populations from regions with low malaria transmission, a certain degree of naturally acquired immunity can develop when the right antibody subclasses are produced. This provides important insight into the potential mechanisms regulating protective immunity.

**Electronic supplementary material:**

The online version of this article (10.1186/s12936-018-2471-6) contains supplementary material, which is available to authorized users.

## Background

Malaria is one of the largest causes of morbidity and mortality in tropical and subtropical regions of the world [1]. *Plasmodium falciparum* had an incidence of 24.69% (12,878 cases) in 2017 in the Peruvian Amazon [2], and it is considered one the most lethal specie of *Plasmodium* that affect humans due to its drug resistance and ability to potentially cause severe malaria. Humoral immunity and IgG antibodies play a critical role in combating infection through their ability to reduce parasitaemia and clinical symptoms [3–11]. Cytophilic subclasses of IgG (IgG1 and IgG3) have been considered the most important antibodies in the development of immunity to malaria, as these subclasses are capable of mediating the activation of leukocytes via their binding to FcγRI and FcγRIII. Together, the predominance of these subclasses is associated with lower risks of malaria-related complications in malaria-endemic areas [12–18].

However, the development of immunity to malaria depends on the balance between cytophilic (IgG1 and IgG3) and non-cytophilic IgG antibodies (IgG4), which interfere with the binding of Fcγ receptors with cytophilic antibodies, complicating the immune response [5, 19, 20]. Noticeably, in the presence of the H131 variant in the FcgRIIA receptor, IgG2 has a cytophilic role, whereas the R131 variant does not bind IgG2 [21–23].

Excreted-secreted antigens are fundamental pieces in the host-parasite interaction and are utilized by various parasites to modulate the immune response of the host [24–26]. These antigens have been recognized for their use in serological diagnostics [27–29] and immunizations [30, 31] in many different parasitic species. The excreted-secreted antigens of *P. falciparum* (*Pf*-ESAs) have been shown to be involved in the process of erythrocyte invasion, the activation of antibodies and lymphocytes, and in complications of infection (reviewed in [24]). Notably, their importance in immunological development in Madagascar was evaluated by Chumpitazi et al. [32], who reported that IgG1 responses specific for *Pf*-ESAs were associated with clinical protection, in contrast to *Pf*-ESA-specific IgG4 responses, which had the opposite effect.

In Peru, the transmission and incidence of malaria is much different than what is observed in Africa, and the Peruvian Amazon is a zone of low malaria transmission with a low prevalence of *P. falciparum*. Nevertheless, transmission throughout the region remains persistent due to a large number of asymptomatic infections [33–35]. Such subclinical cases are the product of the development of immunity to repeated infection, with Clark et al. [36] estimating that one *P. falciparum* infection can elicit the production of enough IgG antibodies to persist for over 5 months. However, it remains to be seen whether the responses of the individual IgG subclasses may be different.

Efforts toward malaria elimination and eradication goals have changed global malaria epidemiology, resulting in a substantial decline in global malaria morbidity and mortality [1]. This study analysed the role of IgG subclasses in controlling malaria disease to help advance malaria vaccine development, which should target geographical areas of low transmission. Therefore, this study characterized the subclasses’ response to *P. falciparum* excreted-secreted antigens in symptomatic and asymptomatic carriers within a community in a low-transmission area of the Peruvian Amazon.

## Methods

### Study area and sample collection

The Malaria Immunology and Genetics in the Amazon (MIGIA) project began in 2003 and is composed of a longitudinal cohort of more than 2000 individuals living in communities in southern Iquitos, Peru, in a zone called Zungarococha (18 M 6830379576887). *Plasmodium falciparum* has a prevalence of 2.6%, with 60% of these infections being asymptomatic [33, 35].

This retrospective study used samples collected between 2008 and 2011 (detailed in flowchart in Additional file 1) cross-sectional surveys were performed for *P. falciparum* infection. Active case detection was conducted for asymptomatic *P. falciparum* infection, and passive case detection for symptomatic malaria episodes was performed at the community health center post-infection. The active case detection and passive case detection methods used in this study are described in more detail in Branch et al. [33].

In both case detection methodologies, a detailed epidemiological questionnaire (including fever, chills, headache, diarrhoea, nausea/vomiting or body aches, in the last 48 h) was administered, and a physical examination was conducted by a physician [33, 36]. Axillary temperature was measured using a digital thermometer, and a finger-prick blood sample (500 μl) was collected in a microtube containing EDTA anticoagulant, from which material for the blood smear slide, haematocrit capillary, plasma sample, and red blood cell sample was taken. A demographic survey, GPS coordinates of the home, nutritional examination (if the patients were above the expected height/weight and if they looked healthy) and helminth examination (matched with the Peruvian Ministry of Health [MINSA] malaria species-specific treatment records) were recorded for each individual, as described in Branch et al. [33] and Clark et al. [36].

### Parasite culture

The 3D7 strain was cultivated in RPMI 1640 media according to the modified candle-jar technique (5% AlbuMax II) with a mix of gases (5% O_2_, 5% CO_2_, 90% N_2_) [37]. Once 5% parasitaemia was reached, cultivation was synchronized with the Percoll-sorbitol technique. When parasitaemia reached 20%, *Pf*-ESA extraction began.

### *Pf*-ESA extraction

Synchronized cultures with 20% parasitaemia were washed 3 times in incomplete media (RPMI 1640) by centrifugation at 1460×*g* for 2.5 min at room temperature. Red blood cells were resuspended in incomplete media with a haematocrit of 5% and were incubated for 24 h at 37 °C. After incubation, the supernatant was centrifuged at 2000×*g* for 5 min at room temperature. The supernatant was collected and underwent further centrifugation at 7000×*g* for 30 min at 4 °C. Protein quantification was carried out according to the Bradford method [38].

From the incubation process, three antigens were obtained: (i) ring phase (first 24 h of the parasite’s biological cycle); (ii) trophozoite-schizont phase (between hour 25 and hour 48 of the parasite’s biological cycle) and (iii) *Pf*-ESAs, a mixture of antigens (i) and (ii) in a volumetric ratio of 1/1. In the standardization process described in Saavedra-Langer [39], it was determined that antigen (iii) presents the best immunoreactivity in comparison to antigens (i) and (ii). Based on these results, *Pf*-ESA was used to characterize the whole IgG and subclass responses in symptomatic and asymptomatic carriers.

### Plasma samples

Based on the epidemiological questionnaire and the physician’s examination, the positive carriers were classified into two groups. Symptomatic patients were defined by parasites identified via blood smear and PCR as well as one of the following: an axillary temperature of ≥ 38.3 °C or a self-reported history of fever within 2 days prior to diagnosis, or a haematocrit of < 30% PCV [36]. Asymptomatic patients did not present with fever or any symptoms (fever, chills, headache, diarrhoea, nausea/vomiting or body aches) in the last 48 h but tested positive by microscopy and PCR. All samples were collected on the day of diagnosis, Day 0.

### Measurement of IgG titers

Enzyme-linked immunosorbent assays (ELISAs) were used for the detection of IgG antibodies according to previously described protocols [36]. *Pf*-ESAs were diluted with borate-buffered saline (BBS) to a final concentration of 1.35 µg/ml and were added to immunosorbent plates (Nunc) before being incubated overnight at 4 °C. The following day, they were washed once with phosphate-buffered saline (pH 7.4) containing 0.05% Tween-20 (PBS-T). The plates were then blocked with blocking solution (PBS containing 1.5% skim milk) for 1 h before being washed three times with PBS-T. Plasma was diluted 1:200 with AB washing solution (0.15 M Na_2_HPO_4_, 0.05% Tween-20, 0.05% BSA, 500 mM NaCl) containing 0.5% skim milk before being added to the plate in duplicate and incubated for 1 h at room temperature. The wells were washed four times with AB washing solution to remove any residual antibodies. Next, 1:3000 dilutions of HRP-conjugated goat anti-human secondary antibodies (Southern Biotech) were incubated with the plates for 1 h at room temperature to detect any antibodies that had bound to the *Pf*-ESAs. After incubation, the plates were washed three times with AB washing solution to remove any remaining secondary antibodies. Then, 50 µl of 3,3′, 5,5′-tetramethylbenzidine (Sigma) was added to the wells to detect the presence of secondary antibodies. After 1 h of incubation, this reaction was stopped via the addition of 25 µl of 0.25 M HCl. The optical densities (ODs) were determined at 450 nm using a plate reader (OSYS MR DINEX). All samples were evaluated in duplicate with coefficients of variation below 25%. Each plate had a pool of positive control sera composed of 10 samples positive for *Pf*-ESA-specific IgGs and were diluted at set ratios between 1:50 and 1:102,400, as per the protocol described by Clark et al. [36], as well as a pool of negative controls from healthy residents of Peru and the USA (n = 10) who had never been exposed to malaria.

To standardize data obtained from different plates, arbitrary ELISA AU was determined by generating a standard curve using serially diluted positive control sera in each plate [40]. Was used the reciprocal of the dilution of the endpoint titer times each dilution to assign AU to the standards and then fit the data to a symmetrical four-parameter log-logistic model [41]. Using inverse regression, the AU of each sample was estimated based on the mean OD of duplicates per plate.

The seroprevalence in the sample for total IgG and each subclass were determined using a mixture model fit to the AU distribution. This assumed that the samples were composed of a mixture of latent seronegative (*S*^−^) or seropositive (*S*^+^) populations as more than one Gaussian distribution [42]. The number of components in the model was determined in terms of the lowest proportion of individuals with unclear classification. For a three-component mixture model, the assumption of immunity increasing upon recurrent malaria exposure in certain individuals allowed for the interpretation of the intermediate component as an exposed-seropositive population and the third as a boosted-seropositive population, defining them as *S*^−^ = 1; *S*^+^ = 2, 3. The assignation of each individual to each corresponding serological population was performed using a conditional classification probability of 90%.

### Measurement of the IgG subclasses

The reactivity of IgG1-IgG4 antibody subclass were measured according to procedures already described for total IgG. The secondary antibodies were diluted 1:2000 for both IgG1 (HRP-conjugated rabbit anti-human IgG1, Southern Biotech) and IgG3 (HRP-conjugated rabbit anti-human IgG3, Southern Biotech) and were diluted 1:1000 for both IgG2 (HRP-conjugated rabbit anti-human IgG2, Southern Biotech) and IgG4 (HRP-conjugated rabbit anti-human IgG4, Southern Biotech).

### Data analysis

The differences in AU means between symptomatic patients and asymptomatic carriers for total IgG and each subclass were tested using a Mann–Whitney test and tested the differences in proportions of serological populations in the same groups using Fisher’s exact test. The correlation among antibody levels between groups were assessed with the individual’s parasite density and age using a Spearman correlation test for each group of individuals. For demographic and clinical characteristics, Pearson’s Chi squared test was used for categorical data and a Mann–Whitney test for continuous data. All data analysis was performed using R software version 3.4.2 [43].

## Results

From all the samples collected during cross sectional surveys, were selected 57 samples which present the criteria detailed in “Methods*”*—“Study area and Sample collection*”* and “Plasma samples*”.* Other positive samples not were considered for the lack of some information (see Additional file 2).

### Characteristics of study participants

Among 57 subjects, differences in sex or age between groups did not observe (Table 1). With respect to clinical parameters, the parasite density was significantly higher (P < 0.001) in symptomatic carriers (median of 2735 parasites/µl; interquartile range, 1130–5824 parasites/µl) compared to asymptomatic carriers. Only one symptomatic carrier had a haematocrit of < 30% PCV. Three asymptomatic carriers took self-medication.

**Table 1:** Demographic and clinical characteristics of patients

	Asymptomatic *n *= 28 (%)	Symptomatic *n *= 29 (%)	Total *n *= 57 (%)	*P* value^d^
Sex				0.907
Male	15 (53.6)	17 (58.6)	32 (56.1)	
Female	13 (46.4)	12 (41.4)	25 (43.9)	
Age (years)^a^	26.0 (18.8–37.2)	26.0 (19.0–35.0)	26.0 (19.0–36.0)	0.975^e^
Parasite density (/µl)^a^	405 (0–1856)	2735 (1130–5824)	1171 (294–4323)	< 0.001^e^
Helminths				0.506
No	18 (64.3)	22 (75.9)	40 (70.2)	
Yes	10 (35.7)	7 (24.1)	17 (29.8)	
Self-medication				< 0.001
No	25 (89.3)	9 (31.0)	34 (59.6)	
Antibiotics	1 (3.57)	0 (0.00)	1 (1.75)	
Anti-inflammatory	2 (7.14)	1 (3.45)	3 (5.26)	
Antipyretic	0 (0.00)	19 (65.5)	19 (33.3)	
PCV^b^	39.1 ± 3.57	39.2 ± 4.30	39.1 ± 3.93	0.950^f^
Community				0.890
Zungarococha	7 (25.0)	5 (17.2)	12 (21.1)	
Puerto Almendra	5 (17.9)	6 (20.7)	11 (19.3)	
Nina Rumi	8 (28.6)	8 (27.6)	16 (19.3)	
Llancham	8 (28.6)	10 (34.5)	18 (31.6)	
Above the expected height/weight^c^				
No	0 (0.0)	0 (0.0)	0 (0.0)	
Yes	28 (100.0)	29 (100)	57 (100)	
Looks healthy^c^				0.201
No	3 (10.7)	8 (27.6)	11 (19.3)	
Yes	25 (89.3)	21 (72.4)	46 (80.7)	

^a^Non-normally distributed numerical variables: median (interquartile range)

^b^PCV (Packed Cell Volume): mean ± standard deviation

^c^Under the perception of a health professional

Tests used: ^d^Pearson’s Chi squared test, ^e^Mann–Whitney test, ^f^T-Student test

### Total antibody responses to Pf-ESAs

**Fig. 1:**
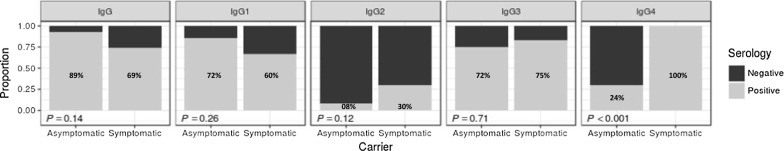
Proportion of serological populations (seronegative, S -, and seropositive, S +) per total IgG and subclass (IgG1, IgG2, IgG3 and IgG4), stratified by asymptomatic or symptomatic carrier. The sample was classificatied in two populations based on a three-component mixture model (defined as S - = 1; S + = 2, 3) to compare the proportions between carriers per each IgG subclass by Fisher’s exact test. The percentages are indicated within the columns

Total IgG antibody responses to *Pf*-ESAs was identified in 79% (45/57) of individuals (Fig. 1). Asymptomatic patients had a higher antibody response compared to symptomatic individuals (P < 0.05) (Table 2), although no difference in the proportion of seropositive samples was found between the asymptomatic (89%, 25/28) and symptomatic (69%, 20/29) groups (Fig. 1).

**Table 2:** Humoral response of IgG antibodies and subclasses to *Plasmodium falciparum* ESA

	Asymptomatic n = 28	Symptomatic n = 29	Total n = 57	*P* value
Ig subclass				
IgG	127.08 (65.10–170.58)	79.12 (9.59–125.65)	109.80 (37.36–139.88)	0.01
IgG1	400 (196–519)	314 (102–532)	391 (140–521)	0.54
IgG2	20.2 (16.2–30.8)	13.6 (11.1–52.6)	21.8 (15.2–34.2)	0.53
IgG3	442.9 (91.1–889.9)	661.6 (11.1–52.6)	636.2 (98.0–1077.8)	0.11
IgG4	17.4 (12.3–23.7)	43.5 (38.3–47.8)	31.8 (15.7–44.1)	< 0.001

Median (interquartile range)

Test used: Mann–Whitney test

*Ig* Immunoglobulin

### IgG subclass responses to ESAs

The IgG3 and IgG1 subclasses had the highest response levels (mean 636.2 and 391.4 AU, respectively). However, no differences were found in either the proportion of seropositive samples or mean antibody responses between the symptomatic and asymptomatic groups. Only in the IgG4 subclass were the proportion of seropositive samples (P < 0.001) and the mean antibody response (P < 0.001) significantly higher in symptomatic patients (Table 2 and Fig. 1).

### Relatedness of total IgG and subclasses in symptomatic and asymptomatic carriers

Total IgG responses showed a high degree of correlation with both IgG1 and IgG3 for all samples (P < 0.001 each) (Table 3). Because the antibody responses exhibited some variability between the symptomatic and asymptomatic groups, the correlations between total IgG and the subclasses within each population were compared (Table 3). The correlation between IgG1 and IgG3 was higher in asymptomatic carriers (P < 0.001) than in symptomatic patients (non-significant). In contrast, the correlation between IgG2 and total IgG (P < 0.001), IgG3 (P < 0.001), and IgG4 (P < 0.01) was higher in symptomatic patients compared with the other group of patients.

**Table 3:** Correlation between total IgG and subclass (IgG1, IgG2, IgG3 and IgG4) response levels to *Pf*-ESAs stratified by asymptomatic or symptomatic carriers

	IgG1	IgG2	IgG3	IgG4
All individuals				
IgG	0.53 (***)	0.45 (**)	0.52 (***)	− 0.14 (ns)
IgG1		0.23 (ns)	0.47 (**)	0.14 (ns)
IgG2			0.68 (***)	0.24 (ns)
IgG3				0.38 (**)
Asymptomatic				
IgG	0.56 (**)	0.32 (ns)	0.57 (**)	− 0.09 (ns)
IgG1		0.07 (ns)	0.63 (***)	0.32 (ns)
IgG2			0.40 (*)	0.10 (ns)
IgG3				0.29 (ns)
Symptomatic				
IgG	0.52 (*)	0.77 (***)	0.68 (***)	0.33 (ns)
IgG1		0.34 (ns)	0.36 (ns)	0.22 (ns)
IgG2			0.90 (***)	0.57 (**)
IgG3				0.50 (*)

Correlation coefficients were determined by Spearman’s method

*ns* non-significant

* P < 0.05; ** P < 0.01; *** P < 0.001

### Correlation of total IgG and subclasses with parasite density

**Fig. 2:**
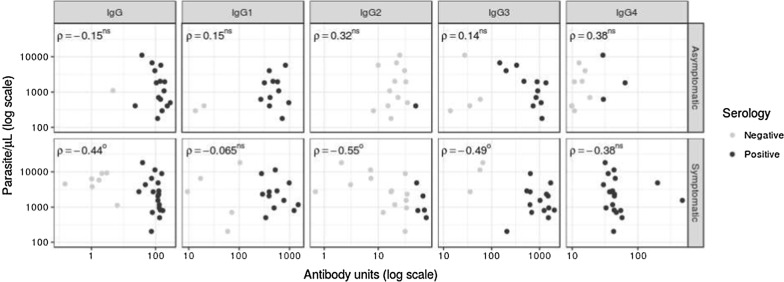
Correlation between parasite density with total IgG and subclass response levels stratified by asymptomatic or symptomatic carrier. Correlation coefficients were determined by Spearman’s method. We show data for individuals as a dotplot. Superscripts denote ^O^P < 0.05; ^OO^P < 0.01; *ns* non-significance

For symptomatic patients, the parasite density was negatively correlated with total IgG (P < 0.005), IgG2 (P < 0.05), and IgG3 (P < 0.05). In contrast, antibody responses of asymptomatic patients did not have any correlation with the patients’ levels of parasitaemia (Fig. 2).

### Correlation of total IgG and subclasses with age

**Fig. 3:**
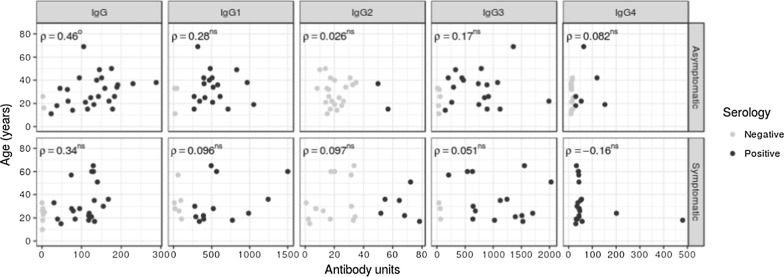
Polarization of total IgG and subclass response levels with respect to age, stratified by asymptomatic or symptomatic carrier. Correlation coefficients were determined by Spearman’s method. We show data for individuals as a dotplot. Superscripts denote ^O^*P *< 0.05; ^OO^*P *< 0.01; *ns* non-significance

Only in asymptomatic patients, age was positively correlated only with the total IgG antibody response (P < 0.05, respectively) (Fig. 3).

## Discussion

Understanding the nature of immunity to malaria is essential for controlling and eradicating the disease. This is especially true in areas of low transmission, such as the Peruvian Amazon, where despite the low prevalence of *P. falciparum*, the ability to develop protective immunity has been demonstrated [33–36]. Indeed, the high incidence of asymptomatic cases and lack of complications associated with symptomatic infections help facilitate sustained transmission of malaria in the region, thereby complicating control efforts.

These results indicate that the levels of whole IgG are higher in asymptomatic carriers (P < 0.05), similar to results from other studies [3–6, 9, 10, 20, 44–48], which showed that higher levels of IgG antibodies were associated with protection against the complications of malaria. Additionally, these carriers have a stronger correlation between IgG1 and IgG3 (Table 3), a common association in communities of low malaria transmission [16].

Both carriers (symptomatic and asymptomatic) show a predominance of IgG3 and IgG1 responses (Fig. 1) in comparison to other studies that used exoantigens, such as Chumpitazi et al. [32] and Ferreira et al. [49], who observed a predominance of IgG2 in their populations; it is important to clarify that these studies only analysed the whole population without discrimination of symptomatology. These results reflect the heterogeneity of the antibody response in malaria infections, as described previously [15, 49–51].

Although asymptomatic antibodies are characterized by higher titres of cytophilic antibodies [5, 10, 20, 45, 46], differences in the titres of cytophilic antibodies between both carriers were not observed (Fig. 2), in contrast to reports by Scopel et al. [52] and Braga et al. [45] for IgG3, which was present at higher levels in both carriers (661.6 and 636.2) (Table 2). Medeiros et al. [6] did not observe differences in the responses to the recombinant antigens MSP1-19kD and MSP3-3D7. A stronger correlation was observed between IgG2 and IgG3 (Table 3), which is especially notable because these subclasses had a negative correlation with parasitaemia only in symptomatic carriers (Fig. 3). Although IgG2 was not present at high levels in this study (Table 2 and Fig. 1), it can provide protection, as observed in other studies [22, 32, 49, 53]. However, to elucidate its activity, the authors suggest evaluating the Zungarococha population for an H131 polymorphism that may be related to the protective activities of IgG2.

These results can explain why symptomatic carriers in the Zungarococha community do not show severe malaria complications (neurological symptoms, severe anemia, or respiratory distress), as observed by Branch et al. [33] and did not have cases of hyperparasitemia (> 100,000 parasites/μl), which is consistent with the observed median parasitaemia of 2735 parasites/µl (Table 1).

As Medeiros et al. [6] have suggested, it can be assumed that symptomatic carriers may be in the process of acquiring clinical immunity to malaria and will become asymptomatic in the future. Part of this process of acquiring clinical protection requires a balance between cytophilic and non-cytophilic responses. However, this process is not at all complete in symptomatic carriers, as the results reflect higher antibody levels and seroprevalence of IgG4 in symptomatic carriers. The binding of IgG4 to an antigen blocks the recognition of the antigen by cytophilic IgG and, therefore, the activation of effector cells through Fc receptors [4, 22, 51, 54–56]. This imbalance in the major mechanism controlling malaria symptoms is reflected in the fever, chills, nausea, and body aches observed in Zungarococha settlers [33].

The principal limitation of this study was the number of samples (n = 57), limiting factors in the measure of co-variables related to nutritional status (Table 1) and lack of specific demographic and clinical information to increase the number of samples (Additional file 2).

But in these circumstances, a high cytophilic antibody response was observed in this community, and symptomatic carriers were characterized by the IgG4 response. Further studies utilizing larger sample sizes will be required to confirm these results, especially the role of IgG4 in clinical symptoms, elucidating its role as a marker of susceptibility that helps to identify clinically relevant patients in Zungarococha. In addition, such studies could clarify the pro-inflammatory response in these patients and help to elucidate the factors involved in the regulation of the immune response in both asymptomatic and symptomatic carriers. Such efforts would ideally evaluate the IgG subclass responses during both the seasons of highest and lowest transmission to determine the temporal effects on antibody titers and to identify the duration of each immune response.

Notably, the *Pf*-ESAs elicited elevated seropositivity for two cytophilic antibodies, resulting in a response similar to those antigens used by Garraud et al. [57]. This serves to reinforce their importance in the development of protective immunity as well as their possible role in vaccine development. Furthermore, as *P. falciparum* shows a high genetic diversity in the study area [58], wild strains may present different antigens, as was described in Anders et al. [59], so that is important analyse antibodies responses to *Pf*-ESAs obtained from wild strains isolated in Zungarococha community. Also, serological techniques, such as Western blots would allow us to determine the protein profiles of *Pf*-ESAs for each antibody subclass, similar to studies by Olesen et al. [20] and Ouevray et al. [60]. This could help us to determine the differences in immunity between asymptomatic and symptomatic individuals and to identify possible markers, thereby improving the understanding of the changes associated with the development of immunological protection against clinical disease.

## Conclusion

The Peruvian Amazon requires more research to help understand the immune response to malaria in populations with a high prevalence of asymptomatic and low-density infections. This is the first study that characterizes the IgG subclass response in the Peruvian Amazon, and these results show that populations from regions with low malaria transmission can develop an appropriate cytophilic response by IgG subclass antibodies, while symptomatic carriers require non-cytophilic responses to develop protective immunity. Additionally, these findings can contribute to a better understanding of immunity in populations exposed to malaria transmission that could be beneficial in the development and testing of *Pf*-ESA-based vaccines.

Further studies are needed to evaluate whether the IgG subclass responses are better markers of protective immunity than total IgG responses and to understand their role in protection, especially against other antigens that are considered to have a potential use in vaccination.

## Additional files

**Additional file 1.** Flowchart of sample selection.

**Additional file 2.** Raw data of the AU, OD, parasitaemia and characteristics of study participants.

## Abbreviations

AU: antibody units
ESAs: excreted-secreted antigens
FcγRI: Fc-gamma receptor 2
FcγRII: Fc-gamma receptor 2
IgG: immunoglobulin G
OD: optical density
