# Supplementary figures and images for: IgG subclass responses to excreted-secreted antigens of Plasmodium falciparum in a low-transmission malaria area of the Peruvian Amazon
Source: Malar J. 2018 Sep 11;17:328. doi: 10.1186/s12936-018-2471-6 (PMC6131892; doi:10.1186/s12936-018-2471-6)

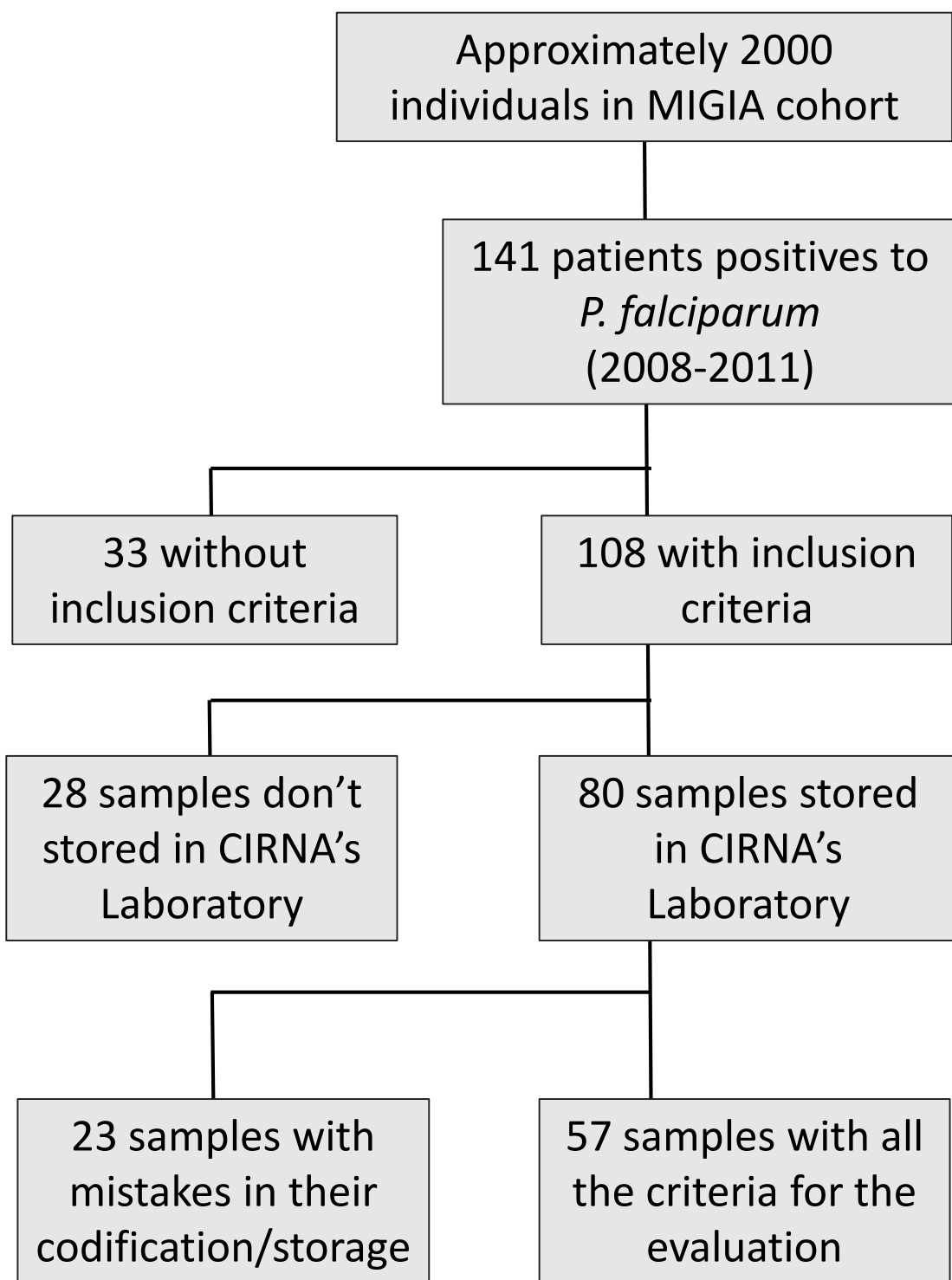

Flowchart of sample selection.

Supplement: Supplementary file 1 — Additional file 1. Flowchart of sample selection. [file 12936_2018_2471_MOESM1_ESM.pdf]
